# Fully 3D Printed Tin Selenide (SnSe) Thermoelectric Generators with Alternating *n*-Type and *p-*Type Legs

**DOI:** 10.1021/acsaem.3c00576

**Published:** 2023-05-03

**Authors:** Matthew Richard Burton, Geraint Howells, Shahin Mehraban, James D. McGettrick, Nicholas Lavery, Matthew J. Carnie

**Affiliations:** † SPECIFIC-IKC, Department of Materials Science and Engineering, Faculty of Science and Engineering, Swansea University, Bay Campus, Swansea SA1 8EN, United Kingdom; ‡ MACH 1, Faculty of Science and Engineering, Swansea University, Bay Campus, Swansea SA1 8EN, United Kingdom

**Keywords:** thermoelectrics, printed, tin selenide, thermoelectric generator, TEG, *n*-type

## Abstract

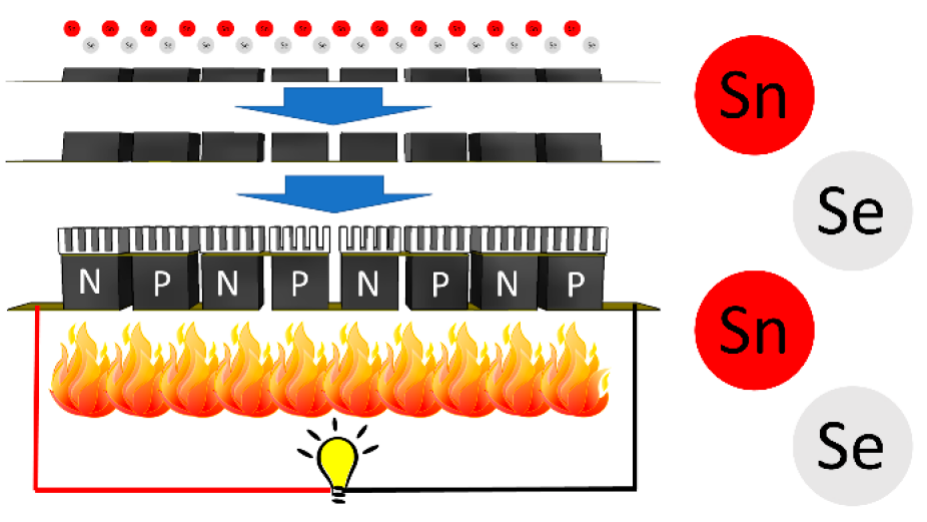

Tin selenide (SnSe) has attracted much attention in the field of thermoelectrics since the discovery of the record figure of merit (*zT*) of 2.6 ± 0.3. While there have been many publications on *p-*type SnSe, to manufacture efficient SnSe thermoelectric generators, an*n-*type is also required. Publications on *n-*type SnSe, however, are limited. This paper reports a pseudo-3D-printing technique to fabricate bulk *n-*type SnSe elements, by utilizing Bi as a dopant. Various Bi doping levels are investigated and characterized over a wide range of temperatures and through multiple thermal cycles. Stable *n-*type SnSe elements are then combined with printed *p-*type SnSe elements to fabricate a fully printed alternating *n-* and *p-*type thermoelectric generator, which is shown to produce 145 μW at 774 K.

## Introduction

In a world that is becoming ever more concerned with climate change and with an ever-dwindling supply of fossil fuels, there needs to be a transition toward green and sustainable energy sources. A 2014 study found that from UK industry alone, 48 TWh yr^–1^ of energy is lost as waste heat.^1^ Harvesting 1% of this wasted heat energy would equate to a reduction of >0.25 Tg yr^–1^ in CO_2_ emissions,^2^ helping meet climate change targets while increasing energy efficiencies. Thermoelectric generators are solid-state devices that are highly reliable and require no maintenance and that offer a route to generate electricity from these waste heat sources by exploiting the temperature gradients they generate.

A thermoelectric generator consists of alternating *n-* and *p-*type materials, connected electrically in series and thermally in parallel, sandwiched between an electrical insulator with high thermal conductivity (e.g., alumina). The ability of a device to harness heat energy can be compared with the figure of merit for a device (*ZT*), whereas the ability of thermoelectric materials to harness heat energy at any given temperature (*T*) is compared by the materials figure of merit (*zT*), as shown in eq 1. The higher the *zT* value, the more efficient the thermoelectric material is; this leads to a desire to increase the Seebeck coefficient (*S*) and electrical conductivity (σ) while simultaneously lowering the thermal conductivity (κ). The transport characteristics (σ, *S*, κ), however, depend on interrelated material properties;^3^ optimizing one variable conflicts with another. One example of this is the Wiedemann–Franz law, which states that the ratio of electrical conductivity (σ) to the electrical component of thermal conductivity (κ_e_) is proportional to temperature. This means an increase in σ leads to an increase in thermal conductivity (κ) and limits the tuning of thermal conductivity to the lattice component κ_L_ (κ = κ_e_ + κ_L_).
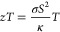
1

Due to the parameters within *zT* being interrelated, there had not been a substantial increase in *zT* since the 1950s, when a *zT* value of around 1 was discovered in Bi_2_Te_3_ and PbTe.^4,5^ In the past decade, however, significant improvements in *zT* have been reported for materials such as Cu_2_S_0.52_Te_0.48_ (*zT* of 2.1), PbTe-SrTe (*zT* of 2.5), and SnSe (*zT* of 2.6).^6−8^ While these values present a significant step forward in thermoelectric materials, they are all *p-*type materials. For efficient thermoelectric generators to be produced, high *zT* value *n-*type materials are also required. While high *zT* value *n-*type materials are limited in the literature,^9−12^ a high *zT* was measured in SnSe that had been doped with Bi (2%, 4%, and 6%) to turn SnSe from *p-* to *n-*type; the added Bi was found to take Sn sites.^13^ Bulk SnSe at 300 K has an orthorhombic crystal structure of the *Pnma* phase (*a* = 11.49 Å, *b* = 4.44 Å, *c* = 4.14 Å), while at high temperatures (750–800 K) SnSe adopts a *Cmcm* structure phase.^14,15^ Bi-doped SnSe at room temperature has been shown to maintain the orthorhombic structure with space group *Pnma* with an *a* axis lattice constant of 11.483 Å.^13^ It is also of note that SnSe is a material of much interest in the fields of photovoltaics (PV),^16^ Li-ion and Na-ion batteries,^17^ and supercapacitors.^18^

The recent increases in *zT* are significant; however, a *zT* of 1 can yield efficiencies comparable to those of other renewable technologies.^19^ While solar and wind renewable technologies have become universal among nations aiming to become carbon neutral, thermoelectrics are currently only being utilized in niche applications, such as space, watches, and Peltier coolers.^20−22^ This can partly be rationalized when considering the high manufacturing costs for thermoelectric generators, which contributes to the relatively high energy cost of thermoelectric generators ($0.80 kWh^–1^),^23^ compared to PV ($0.089 kWh^–1^) and wind turbines (£0.084 kWh^–1^).^24^ One way to lower the cost of thermoelectric generator manufacturing is to use printing.^25^ In contrast to the current commercial manufacturing techniques for thermoelectrics (e.g., spark plasma sintering, followed by manual module assembly), printing can be done at low temperature and pressure and achieves rapid manufacturing through automation. In addition, printing can allow for bespoke elements to be produced that meet the curvature of a waste heat source (e.g., a pipe), unlike the current high-pressure manufacturing techniques that lead to flat elements being produced. While the printing of thermoelectrics has been studied for decades, the advent of 3D-printed thermoelectrics in the last five years has allowed for the realization of *zT* values in excess of 1 and for the manufacture of element sizes required for efficient thermoelectric generators to be produced.^26−31^

The highest *zT* value reported to date for a printed material was that of *p-*type SnSe (*zT* of 1.7).^26^ In this paper, we investigate the addition of four different Bi concentrations to the initial Sn and Se powders before mechanical alloying in an attempt to achieve printed *n-*type SnSe. The thermoelectric and material properties are studied over a wide temperature range, and the thermal stability of samples is studied. Finally, we manufacture and characterize a fully printed *n-* and *p-*type SnSe thermoelectric generator.

## Experimental Section

### Ink Formulation

Sn (≥99%, Sigma-Aldrich), Se (≥99.5%, Sigma-Aldrich), and Bi (≥99%, Sigma-Aldrich) powders were placed in a 250 mL stainless steel grinding bowl (Fritsch). Bi doping levels of 2, 4, 6, and 8 wt % Bi compared to Sn were chosen (note Bi powder added and not substituted for Sn powder), which corresponds to empirical formulas of SnSeBi_*X*_ (*X* = 0.0114, 0.0227, 0.0340, 0.0454). In the grinding bowl were placed 30 stainless steel ball bearings 10 mm in diameter. This was secured into a planetary mill (PULVERISETTE 5/4) which was balanced with another filled grinding bowl. This was set to spin (200 rpm for 30 min) followed by a resting period (30 min); this was repeated 60 times with the spin direction changing after every rest period. Binder solutions were made by mixing sodium carboxymethylcellulose (average MW ≈ 250000, Sigma-Aldrich) with deionized water in a weight ratio of 96:4. The binder mixing process was accelerated with the use of a centrifugal mixer (SpeedMixer DAC 150.1 FVZ). Thirteen grams of the binder solution was then mixed with 37 g of a ball-milled powder, with the aid of the centrifugal mixer, a vortex genie, and stirring with a spatula by hand to make a homogeneous ink.

### 3D-Printing Technique

The printing technique has been described in detail elsewhere.^26^ In short, legs of SnSe were created using a 3D ABS sacrificial mold. The mold was placed on a hot plate at 120 °C to which ink was poured in at about ∼2 mm thickness at a time. Once set, more ink was added. This was done until a leg height of ∼1 cm was achieved. The legs were then cured by heating to 873 K from room temperature at a heating rate of 0.5 K/min, before being left to cool naturally.

### Material Characterization

X-ray diffraction (XRD) was performed on a Bruker D8 diffractor with Cu Kα radiation. Scanning electron microscopy (SEM) and energy dispersive X-ray spectroscopy (EDX) were performed on a Joel 7800F FEG SEM with an Oxford Laboratory EDX attachment.

X-ray photoelectron spectroscopy (XPS) and ultraviolet photoelectron spectroscopy (UPS) were performed on a Kratos Axis Supra instrument, and the data were processed by CasaXPS (2.3.24PR1.0). Samples were mounted in electrical contact with the stage to enable a 9 V sample bias during UPS, and the Fermi edge of a clean Ag control was used to calibrate the energy scale to 0 eV. XPS was sampled to a depth of <10 nm using a monochromatic Kα source (225 W, 15 mA) with a footprint of 300 × 700 μm and a pass energy of 20 eV, with Shirley backgrounds and the GL(30) line shape. For UPS the kinetic energy values of the secondary electron cutoff and valence band maximum were determined by fitting tangents using the “step up” and “step down” regions from a He I (21.21 eV) photoemission spectrum with a 10 eV pass energy and 0.05 eV step size. The tangent fitting error specified by the manufacturer is ±0.13 eV.

### Thermoelectric Characterization

Electrical and Seebeck coefficient measurements were conducted using an ULVAC ZEM-3 thermoelectric tester, under a He atmosphere. The uncertainty of electrical conductivity was ±3% and of Seebeck coefficients was ±4%.^32^ Thermal conductivity was calculated with the formula κ = *DC*_p_ρ. Thermal diffusivities (*D*) were determined using a Netzsch 457 LFA instrument with a Netzsch sapphire sample pan and lid for liquid metallic and powder samples, Ø 11 mm × 1.5 mm, and using the Cowon + pulse correction diffusivity model. This was calibrated with a Ø 10 mm Pyroceram 9606 calibration standard. Results are reported in Figures S8 and S9. The uncertainty of thermal diffusivity was ±3%.^32^ Heat capacities (*C*_p_) were deduced from previous research, with an uncertainty of up to ±5%.^8,32,33^ Densities (ρ) were determined using the method of hydrostatic weighing, which uses the Archimedes principle, with results being reported in Figure S10.^34^ Densities were measured both before and after all measurement cycles and all curing conditions had been completed, with no observable changes being discovered. The uncertainty in density measurements was ±1%.^32^

## Results and Discussion

### Chemical and Structural Characterizations

**Figure 1:**
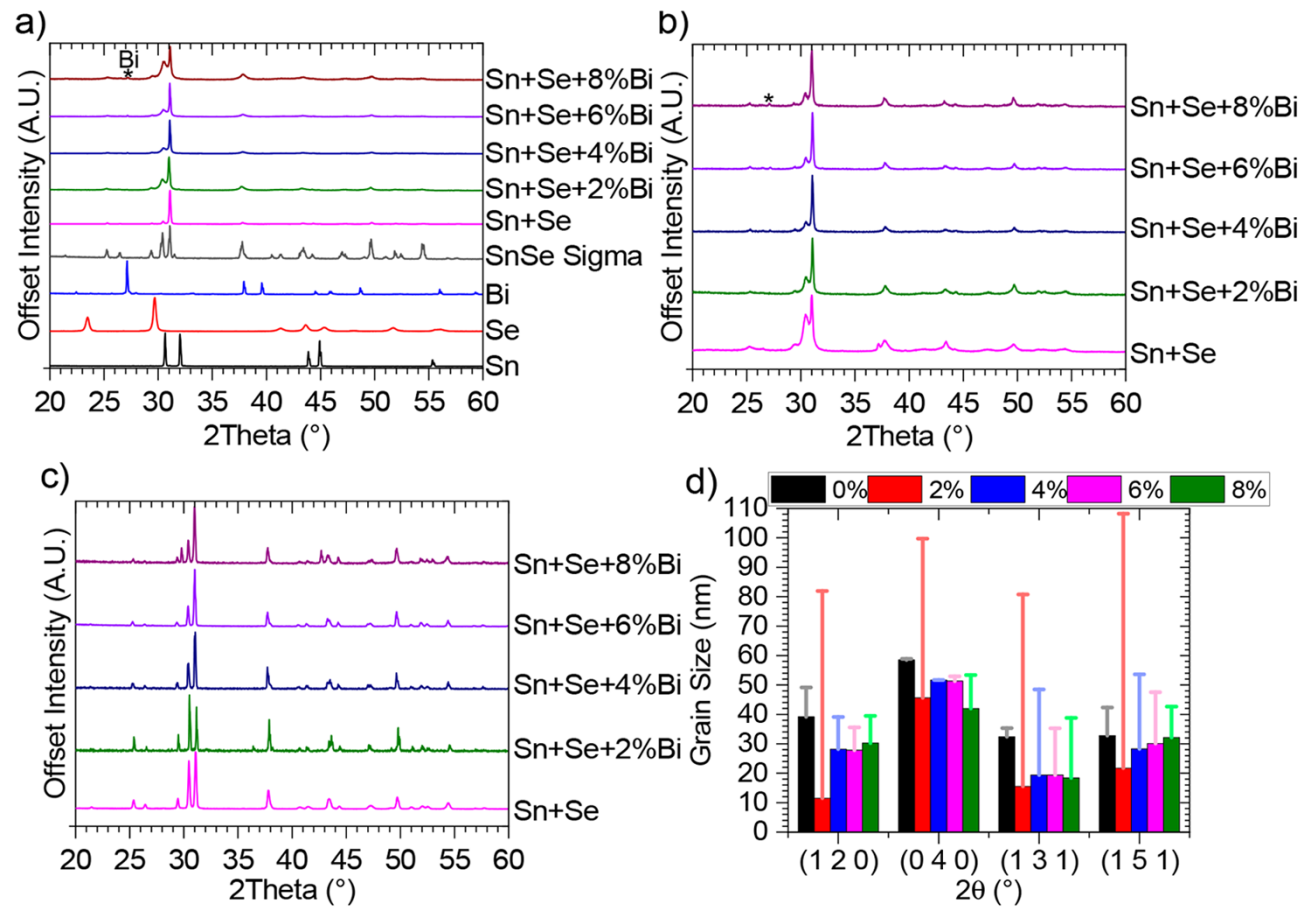
X-ray diffraction: (a) comparison of ball-milled products to starting materials and SnSe (both commercial powder and crystallographic open database ID 1538896);^36^ (b) diffraction patterns of printed samples before cure; (c) diffraction patterns of printed samples post cure; (d) comparison of grain sizes from four peaks, before (columns) and after cure (bars) with all Bi doping concentrations.

To investigate whether the addition of Bi to the Sn and Se powders prior to mechanical alloying (MA) would prevent the formation of SnSe, X-ray diffraction (XRD) of the mechanically alloyed powders along with XRD of the elemental powders was conducted (Figure 1a). All XRD peaks seen for SnSe are seen to be present throughout all Bi doping levels, revealing that the addition of Bi does not inhibit the formation of SnSe through MA. While at 2% Bi doping no peaks from Bi can be seen, further doping does result in a Bi peak at ∼27°. This peak is seen to increase in magnitude with increased Bi doping levels. This reveals that not all the Bi has been incorporated into the SnSe compound above 2% doping levels; however, the small magnitude of the peak and the peak’s absence at 2% Bi doping indicates that a significant proportion of the Bi powder has been incorporated into SnSe. Interestingly, no shift in SnSe peaks are observed when doping with Bi, indicating no change in the lattice parameters. The determined lattice parameters are shown in Table S1 and are consistent with literature values.^8,35^ Normally doping would result in changes to the lattice parameters; however, the highly similar atomic sizes of Bi (1.43 Å) and Sn (1.45 Å) along with Bi occupying Sn sites result in no change of lattice parameters. This is consistent with the literature.^13^ When comparing the diffraction pattern of MA Sn and Se to that of MA Sn, Se, and Bi, the dominance of the (0 4 0) peak is seen to be reduced relative to other peaks seen. This indicates a lower degree of orientation of the powder. In theory a powder pattern should present no orientation; however, powders are not spherical and could pack in a preferred orientation due to powder particle shapes. In addition, when leveling powder flat for a XRD pattern, preferential orientation could also take place. The pattern for MA Sn + Se shows a distinct preferred orientation to the (0 4 0) peak, compared to a commercially sourced SnSe powder (which can be seen in more detail in Figure S1). The addition of Bi prior to MA is seen to lead to a reduction of orientation, which is potentially caused by the formation of more spherical particles. This would be less likely to orient in powder form.

Inks were made by combing MA powders with sodium carboxymethylcellulose and deionized water solutions. These were then printed into sacrificial molds, to leave behind 3D structures.^26^ XRD spectra can be seen for all the printed samples in Figure 1b. Other than a lower level of orientation for the MA pure Sn + Se compared to the powders, no distinct changes can be seen in the patterns. A small peak attributed to Bi can still be seen at ∼27° for samples with >2% Bi doping. Therefore, printing is seen to have no major effects on the powders. XRD patterns were also conducted after the printed samples were cured under a He atmosphere at 873 K (Figure 1c). In these samples the Bi peak (∼27°) is seen to have disappeared, indicating that Bi has now been incorporated into the SnSe compound, there has been Bi loss from these samples, or a combination of the two. After curing the other peaks can be seen to become more intense and less broad, which indicates that the grain size has increased. To assess whether the grain size has changed, the Scherrer equation was used (assuming spherical particles), to calculate the grain size at four separate peaks both before and after the cure. The results can be seen in Figure 1d, with the columns showing the grain size after printing and the bars showing the grain size after curing. On all peaks except for (0 4 0) an increase in grain size is seen, which can be attributed to the temperature effect of the curing process. Bi doping is seen to have very little effect on the crystallite size, except for 2% Bi, which is seen to result in significantly larger grain sizes post curing than for all other samples. This level of doping was also seen to be the only one where no Bi peaks were seen; this may indicate that incorporation of Bi into the SnSe compound may allow grain sizes to increase during curing, but Bi not incorporated into the SnSe compound may inhibit grain size growth.

**Figure 2:**
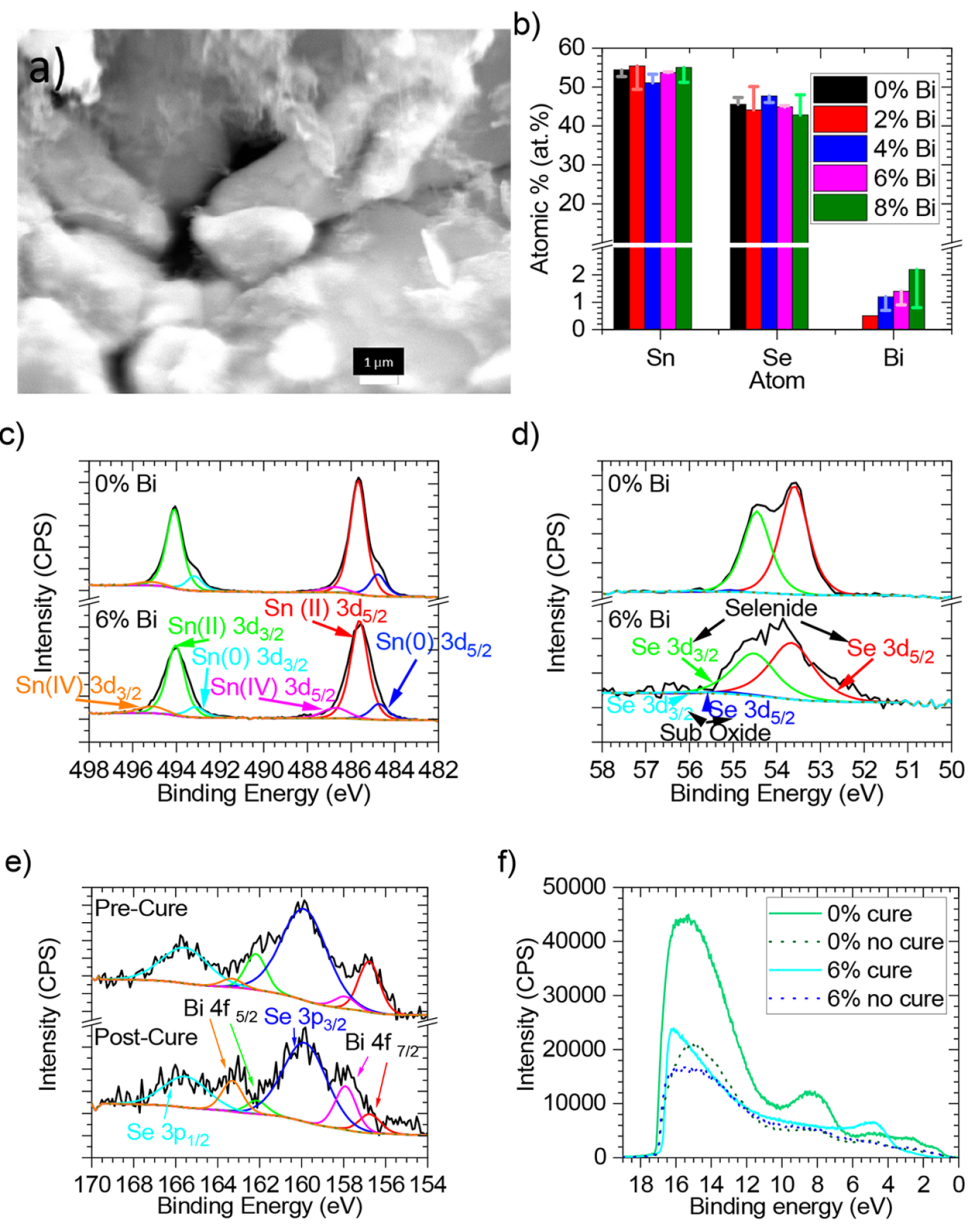
Materials characterization: (a) SEM image of 6% Bi doped cured SnSe; (b) EDX composition of samples before (columns) and after (bars) curing; (c) XPS of the Sn 3d region; (d) XPS of the Se 3d region; (e) XPS of Bi 4f region; (f) UPS of samples before (dotted) and after (solid) cure.

Scanning electron microscopy (SEM) for all samples pre and post cure (Figure S2) also reveals that 2% Bi post cure has significantly larger particle sizes than all other samples. No other discernible differences between the samples can be seen by SEM. An elemental compositional analysis of all samples by energy dispersive X-ray spectroscopy (EDX) can be seen in Figure 2b. All samples are seen to be slightly Sn rich prior to curing, which is believed to be due to evaporation of Se while the inks are drying. This may be due to a small amount of SeO_2_ being formed in the ink, which is soluble in water and could in part evaporate with water from the surface in the drying process.^37^ In contrast, no potential Sn side product is known to be soluble.^38^ Post cure the levels of Sn and Se are seen to be more equal. Pre cure Bi levels are seen to increase as expected with higher percentage additions of Bi, leading to observed percentages that correspond to the empirical formulas. Interestingly, post cure at 873 K all samples above 2% Bi appear to drop to around 1 atom % Bi, while the Bi concentration in the 2% Bi remains unchanged. This, along with the loss of the Bi peak from XRD, indicates that curing removes Bi from these samples. These results do not rule out, however, that curing allows more Bi to infiltrate the SnSe compound, as Bi levels are higher post cure for doping levels above 2% Bi compared to a doping level of 2% Bi.

X-ray photoelectron spectroscopy (XPS) of the Sn 3d region (Figure 2c) reveals that Bi doping has a minimal effect on the Sn oxidation state, albeit with a marginal reduction in Sn(0) and a slight increase in Sn(IV). The same can be said of the Se oxidation state, as highlighted by no change in oxidation states seen in the Se 3d region (Figure 2d). The change in Bi oxidation state can be seen between pre and post cure (Figure 2e). While the same Bi 4f peaks are seen, there is a significant shift to greater oxidation of Bi post cure. This indicates a greater bonding of Bi with SnSe. This shows that the loss of Bi XRD peak post curing is not simply down to a loss of Bi but is also due to greater infiltration of Bi into the SnSe compound. Ultraviolet photoelectron spectroscopy (UPS) was conducted to determine the work function (Figure 2f). Except for 2% Bi doping, no discernible change in work function was seen with Bi content. A mean kinetic energy value for the work function was measured at 4.12 eV (4.09 eV pre and 4.15 eV post cure); this is in line with previously reported density functional theory (DFT) calculations for the work function of α-SnSe monolayers.^39^ At 2% Bi doping a reduced kinetic energy value for the work function was measured, with a value of 3.34 eV being observed post cure (3.94 eV precure). This reduced work function could be due to a different allotrope of SnSe, as this value is broadly in line with DFT calculations for δ-SnSe monolayers.^39^ Alternatively the decrease in work function could be a result of the enlarged grain size seen for 2% Bi. The frain size is seen to affect the work function in many materials,^40−43^ so the same could be true for SnSe. Prior to curing when 2% Bi doped SnSe has a grain size similar to that for other doping concentrations, a similar work function is observed.

### Evaluation of Thermoelectric Properties

**Figure 3:**
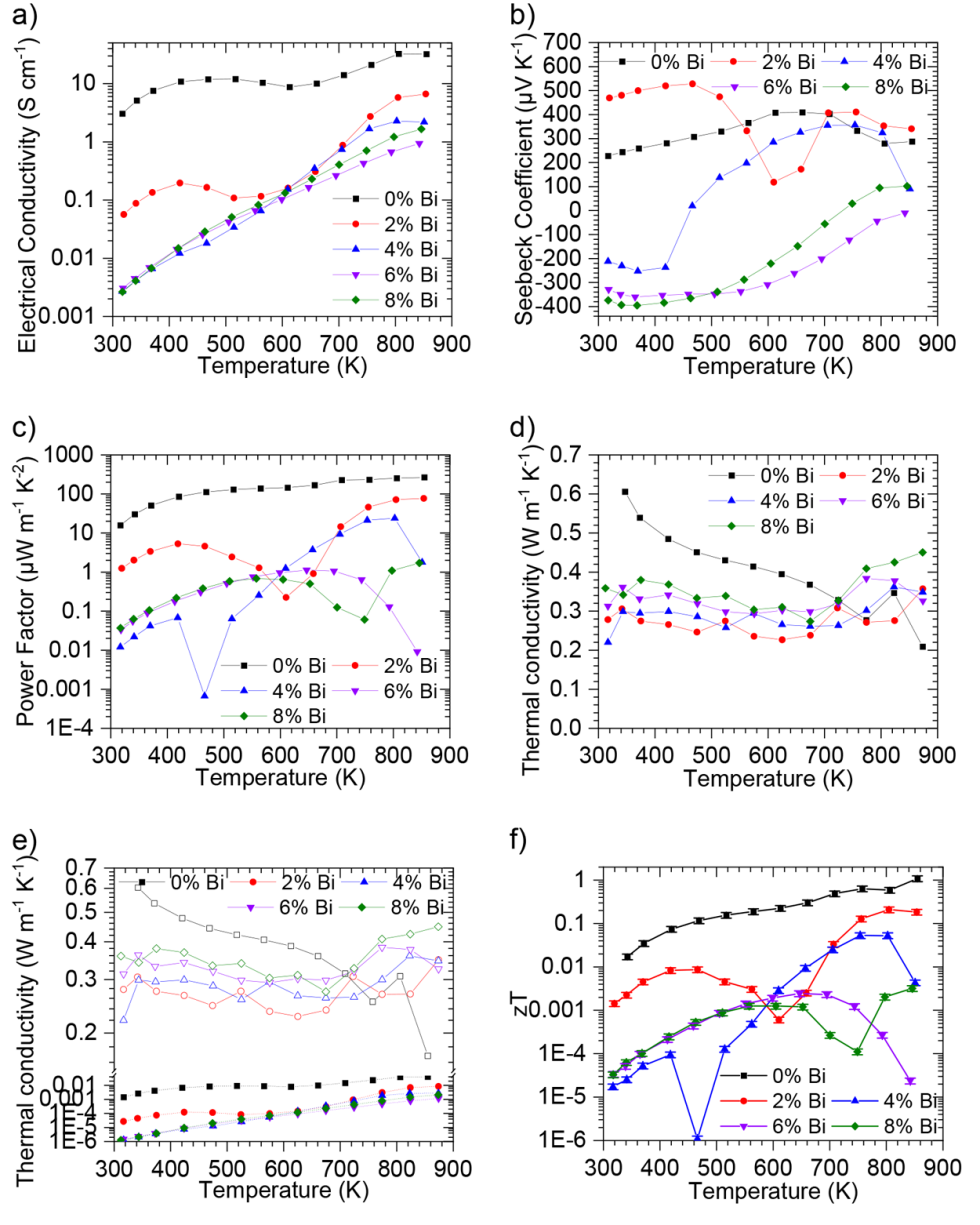
Thermoelectric performance of pseudo-3D-printed SnSe with varying Bi doping levels after curing at 873 K in He: (a) electrical conductivity; (b) Seebeck coefficient; (c) power factor; (d) total thermal conductivity (κ); (e) lattice (empty symbols smf solid lines) thermal conductivity (κ_L_) and electronic (solid symbols and dotted lines) thermal conductivity (κ_e_); and (f) figure of merit (*zT*), with a measurement uncertainty of 15% represented with error bars.^32^

Thermoelectric performance of 2% Bi was unstable during the first two measurement cycles, so for clarity, the thermoelectric performance of samples after they had been measured through two thermal cycles are presented in Figure 3. The first three cycles together can be seen in Figures S3–S7. Electrical conductivity (Figure 3a) is seen to be diminished by the addition of Bi throughout the temperature profile studied, with 2% Bi not being as significant as 4%, 6%, and 8% Bi, which all have similar electrical conductivities. This is in contrast to single-crystal Bi-doped SnSe,^13^ where Bi doping of all concentrations is seen to improve the electrical conductivity and higher Bi doping concentrations (4% and 6%) are seen to improve electrical conductivity more than a lower concentration (2%). In hot-pressed polycrystalline Bi-doped SnSe,^44^ Bi doping was also seen to improve the electrical conductivity perpendicular to the pressing direction; however, Bi doping was seen to reduce electrical conductivity parallel to the pressing direction. Due to the ambient-pressure printing technique resulting in polycrystalline samples (no manufacturing directional bias), these results indicate that the lower electrical conductivity plane is dominant in polycrystalline samples. The Seebeck coefficient (Figure 3b) is seen to vary significantly with Bi doping levels. The Seebeck coefficient for 2% Bi is seen to be consistently *p-*type; however, it is markedly different in magnitude to that of pure SnSe. Based on how the Seebeck coefficient fluctuates with temperature, there appears to be three distinct phases for this printed material. These regions can also be seen in the electrical conductivity (Figure 3a). The initial temperature region up to ∼525 K shows the same temperature dependence as nondoped SnSe; however, as the temperature increases further thermal excitation of carriers is initiated.^8^ This could explain the sudden drop in Seebeck coefficient as more electrons over holes are excited. Equally, the increase in carrier concentration would lead to a drop in Seebeck coefficient, as the two properties are inversely proportional.^3^ Between 600 and 700 K a general increase in Seebeck coefficient is seen. Bi melts at 544 K; therefore, any Bi that is not infiltrated into the SnSe compound could potentially cause a change in carrier concentration. Above 700 K a nearly temperature independent region for Seebeck is seen, which is consistent, albeit at a lower temperature, with observations for nondoped SnSe (which occurs at ∼800 K as also seen in single crystals).^8^ In this region, electrical conductivity is seen to increase.

Electrical conductivity is seen to exponentially increase with temperature throughout the entire temperature range studied for all doping levels above 2% Bi. Bi doping may allow for lower energy thermal excitation of carriers, leading to the observed positive correlation between temperature and electrical conductivity, equally due to the subdued electrical conductivity of Bi-doped SnSe in this work a smaller amount of thermal excitation would cause an increase in electrical conductivity than would be the case for nondoped SnSe. A doping level of 4% Bi is seen to produce a *n-*type material up to 420 K; however, at more elevated temperature the dominant carrier type is seen to change, and the material turns into a *p-*type (during this transition a near-zero Seebeck coefficient is observed, resulting in a near-zero power factor and *zT*). This could be explained by thermal excitation of holes causing a change in the dominant carrier type. In terms of making a stable *n-*type material, 6% and 8% Bi doping shows much greater promise and gives mostly *n-*type product in the temperature range studied. These two higher doping concentrations both show a trend toward a positive Seebeck coefficient with elevated temperatures, suggesting that electrons are becoming the less dominant carrier in the higher temperature regions, and indeed holes are dominant in 8% Bi above 700 K. All power factors for Bi-doped samples (Figure 3c) are lower than those in pure SnSe, with transitions in dominant carrier type reflected in fluctuations of power factor.

Of note is that all densities were stable throughout all measurement and curing cycles and were lower than the theoretical density of SnSe (6.0 g cm^–3^); however, this is not even seen for many single crystals, wherethe density has been seen to be 5.5 g cm^–3^.^8^ Samples in this work have densities ∼80% of this value. More recent work on Pb-doped SnSe managed to achieve around the theoretical density of SnSe;^45^ samples in this work achieve ∼75% of that density. Some of the difference could be explained by differences in preparation methods and handling or measurement errors;^8^ however, most of the difference can be explained by sample porosity. Indeed, printed SnSe has shown thermoelectric performance directly comparable to that of polycrystalline SnSe with a nanoporous design.^26,46^ While Bi doping does result in some change in density (Figure S10), the majority of change in thermal conductivity results from the change in thermal diffusivity. Despite having lower density, the printed samples possess mechanical properties expected for thermoelectric generators. This was tested via drop testing from heights of up to 1 m. All samples remained unchanged from drop tests of all heights tested.

Thermal conductivities (Figure 3d) are for the most part seen to be lower for Bi-doped samples. The thermal conductivity of a material is intrinsically comprised of the sum of the two components, the electronic element (κ_e_) from the carriers and the lattice element (κ_L_) from the phonons. Figure 3e reveals that while some of the decrease in thermal conductivity is due to the electronic component (κ_e_) due to the lower electrical conductivity (κ_e_ = *L*·*T*·σ, assuming *L* = 1.5 × 10^–8^ V^2^ K^–2^),^8^ the majority arises from a decrease in the lattice component (κ_L_). Interestingly, Bi-doped samples show much less variation in thermal conductivity with temperature compared to nondoped samples. This is in contrast to Bi-doped SnSe single crystals.^13^ In hot-pressed polycrystalline Bi-doped SnSe,^44^ the same temperature independence was seen, but only at temperatures in excess of 500 K. Due to the lower electrical conductivities and the smaller Seebeck coefficients at elevated temperatures (where *zT* peaks in SnSe), the *zT* values (Figure 3f) for Bi-doped samples cured at 873 K are seen to be various orders of magnitude lower than that of pure SnSe.

**Figure 4:**
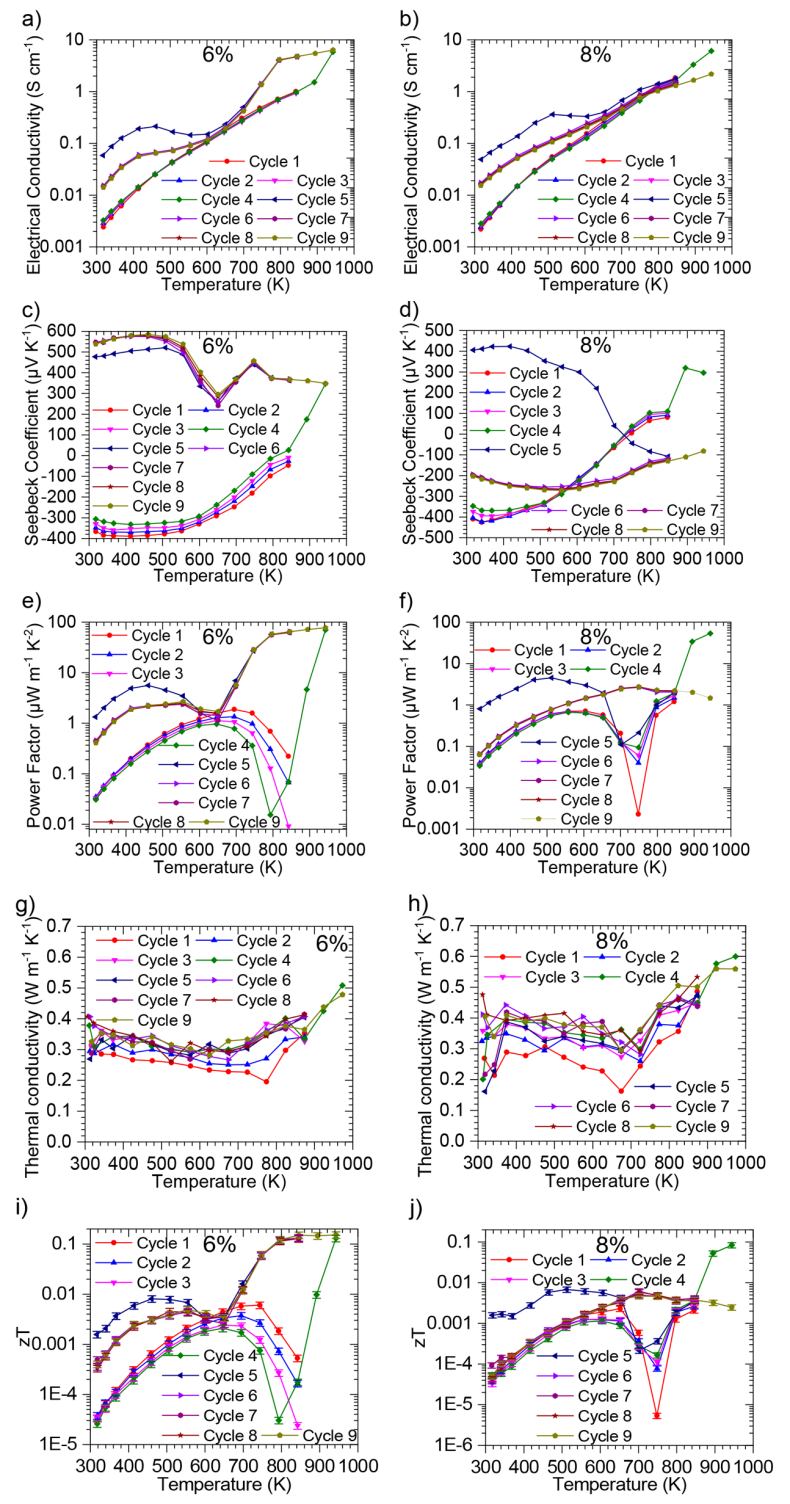
Thermoelectric performance of 6% (a, c, e, g, i) and 8% (b, d, f, h, j) Bi-doped pseudo-3D-printed SnSe after curing at 873 K in He: (a, b) electrical conductivity; (c, d) Seebeck coefficient; (e, f) power factor; (g, h) total thermal conductivity; (i, j) figure of merit (*zT*), with a measurement uncertainty of 15% represented with error bars.^32^

The low *zT* values for Bi-doped printed SnSe samples cured at 873 K can in large part be attributed to the small magnitude of the Seebeck coefficients at elevated temperatures. The low Seebeck coefficient can be explained as being due to the competing factors of the induced *n-*type character from the Bi dopant and the *p-*type character of the intrinsic SnSe. This would potentially suggest that the Bi-doped printed samples were not fully homogenized. To investigate this, the two most promising doping concentrations (6% and 8%) were run through further thermal cycling with the first extra thermal cycle going up to 973 K. The results can be seen in Figure 4. The exposure to a higher temperature is seen to remove any *n-*type character of 6% Bi, with both electrical conductivity and Seebeck coefficients behaving in a manner similar to that of 2% Bi cured at 873 K. This would suggest that the higher cure temperature has resulted in a loss of Bi in the sample. The higher cure temperature is seen to make 8% Bi consistently *n-*type, with much less variability of the Seebeck coefficient with respect to temperature. The magnitude of the Seebeck coefficient is still lower than that of intrinsic printed SnSe, as is the electrical conductivity. These, coupled with slightly higher thermal conductivity values at elevated temperatures, all lead to a lower peak *zT* for 8% Bi-doped printed SnSe when compared to intrinsic printed SnSe. Nonetheless, a stable printed *n-*type SnSe has been achieved through doping with 8% Bi and curing at 973 K, followed by running one thermal cycle to 873 K. No changes in sample dimensions or densities were observed after these thermal cycles, indicating that these could potentially be conducted on fully formed thermoelectric generators and potentially utilizing a waste heat source. The *zT* of this stable *n-*type material is seen to peak at 724 K with a mean value of 0.0054 over the next 4 thermal cycles.

In an effort to improve the thermoelectric properties, the 3D-printing process was adjusted by altering the amount of binder present in the thermoelectric inks. New water to binder weight ratios of 98:2 (2%), 97:3 (3%) ,and 95:5 (5%) were tested alongside the previously tested 96:4 (4%) ratio. The results can be seen in Figure S11. While slight changes in the electrical conductivity and Seebeck coefficients were observed, the power factor remained largely unaffected, with a 96:4 ratio exhibiting the highest average power factor value.

**Figure 5:**
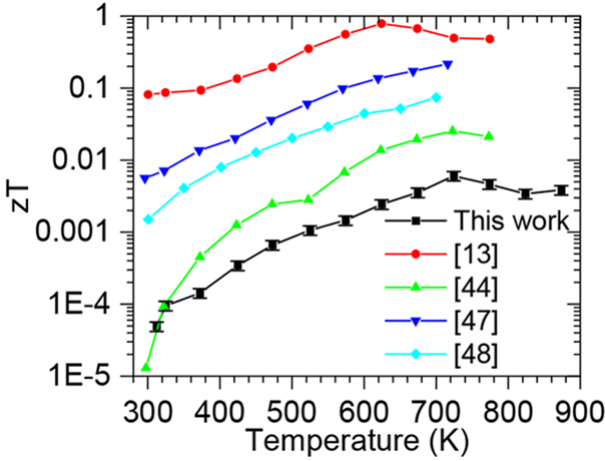
Figure of merit performance of *n-*type pseudo-3D-printed SnSe with 8% Bi doping cured at 973 K, compared to other literature reported *zT* values of Bi-doped *n-*type SnSe.

Compared to other *zT* values reported for Bi-doped SnSe (Figure 5), the values reported in this work are relatively small. The method of manufacturing reported here, however, has both low embodied energy and produces bulk samples. Duong et al.^13^ used higher heating temperatures (1203 K) over a prolonged time (10 h at peak temperature). Nguyen et al.^44^ used the energy intensive method of hot pressing. Chandra et al.^47^ used a low-temperature solution-based route but only produced nanosheets (1.2–3.0 nm thick); they subsequently used spark plasma sintering to make samples large enough to measure the thermoelectric properties. Pang et al.^48^ used chemical vapor deposition and produced thin films.

### 3D-Printed Thermoelectric Device Characterization

**Figure 6:**
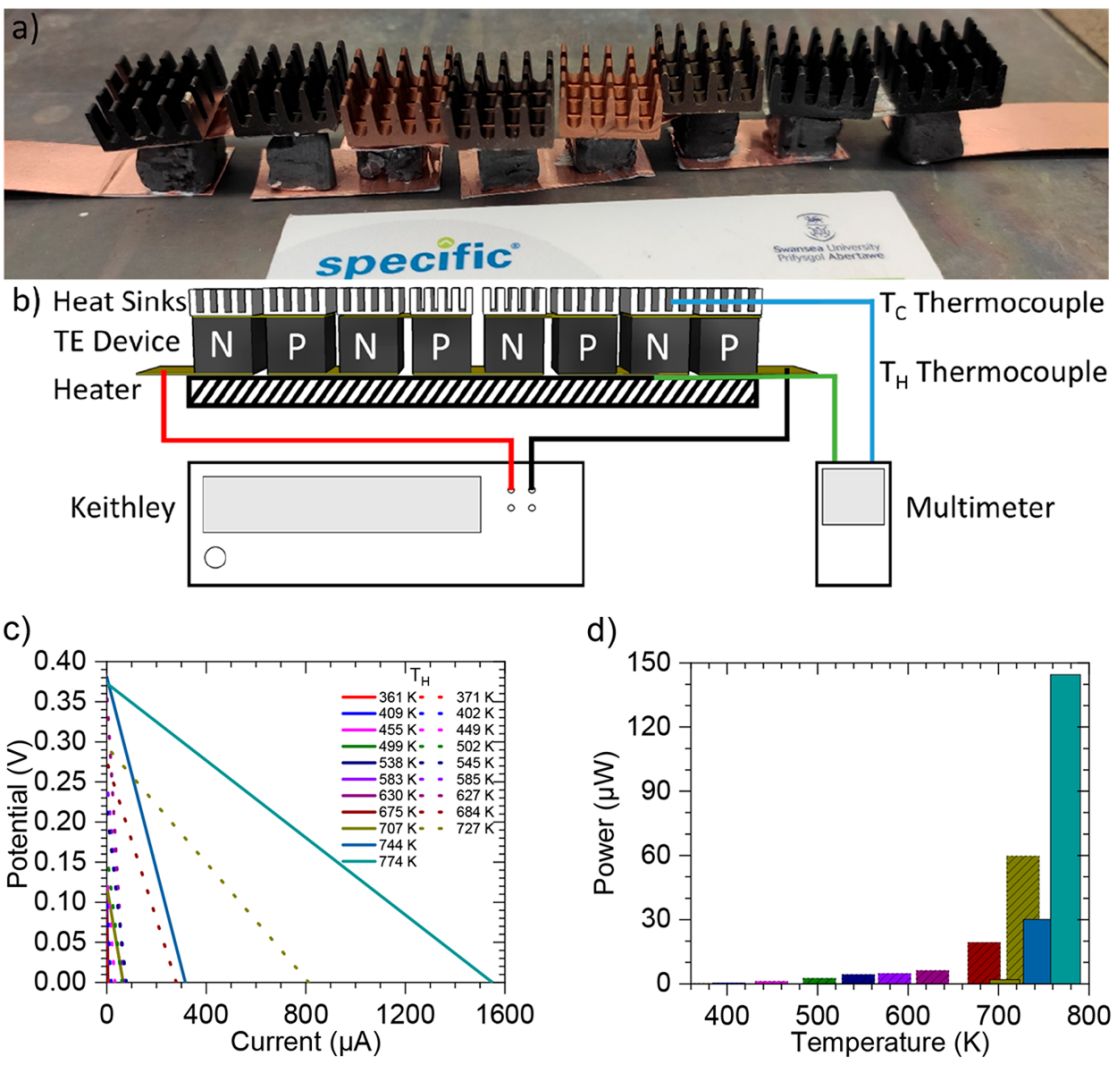
Thermoelectric generator: (a) photograph of the working thermoelectric generator, with alternating *n-* and *p-*type legs; (b) schematic illustration of the device performance setup; (c) the open-circuit voltage (*V*_0_) and short-circuit current (*I*_0_) linearly connected, where solid lines represent values on heating while dotted lines represent values on cooling; (d) peak power outputs of the device (assuming maximum power = *V*_0_*I*_0_/4),^49^ where solid bars represent power on heating while slashed bars represent power on cooling. These data and cold side temperatures can also be found in Table S2 in the Supporting Information.

The stable *n-*type printed SnSe was used to make an all-printed *n-* and *p-*type SnSe 3D thermoelectric generator (Figure 6a), using alternating 3D-printed thermoelectric legs made from 8% Bi-doped SnSe (cured in Ar at 973 K for 30 min) and nondoped SnSe (cured in Ar at 873 K for 30 min). 3D-printed legs were electrically connected in series with Cu, with a layer of Ag paint used to improve electric contacts. Aluminum heat sinks (14 mm × 14 mm × 7 mm) were attached to the top of each leg. A schematic diagram of how the thermoelectric performance of the device was characterized can be seen in Figure 6b. The open circuit voltage (*V*_0_) and the short circuit current (*I*_0_) can be seen in Figure 6c, and the resulting power output of the device can be seen in Figure 6d (assuming maximum power = *V*_0_*I*_0_/4^49^). The power of the device can be seen to increase exponentially with increasing hot side temperature (*T*_H_), with the device reaching 145 μW at the maximum temperature investigated (774 K). The performance of the device on cooling was also measured, which is seen to be enhanced compared to heating. This can be explained due to the enhanced electrical conductivity of SnSe at elevated temperatures; so while cooling the device gave a lower Δ*T*, the performance was enhanced, as the cold side had an enhanced electrical conductivity on cooling compared to heating. There is potential for increased power output through optimization of the device, such as optimizing contacts, leg dimensions, and heat sinks. The maximum hot temperature reached was also significantly below the peak *zT* of the SnSe inks. Nevertheless, the maximum measured power output of 145 μW is a significant improvement from the 20 μW seen for a *p-*type only device,^26^ which we believe represents a significant step toward commercialization of the technology.

## Conclusions

Bi-doped SnSe samples were made with doping levels of 2, 4, 6, and 8 wt % Bi compared to Sn, corresponding to empirical formulas of SnSeBi_*X*_ (*X* = 0.0114, 0.0227, 0.0340, 0.0454). Thermoelectric legs were manufactured using a 3D-printing approach which combines the use of sacrificial printed molds with a layer-by-layer deposition of SnSeBi_*X*_ mixed with a carboxymethylcellulose binder. Curing at 873 K under a He atmosphere was conducted to enhance the thermoelectric performance of the printed samples. The results show that while 2% and 4% Bi were not sufficient to establish any significant *n-*type character, 6% and 8% Bi both revealed *n-*type properties which faded with increasing temperature. Curing of these higher doping levels at a higher cure temperature (973 K) resulted in a loss of *n-*type character in 6% Bi but formed stable *n-*type characteristics in 8% Bi doping. A mean *zT* peak of 0.0054 at 724 K was observed over four thermal measuring cycles. An eight-leg fully 3D printed thermoelectric generator was produced with alternating legs of this stable printed *n-*type material and nondoped printed SnSe legs. A power output of 145 μW was observed, which is over 7 times the measured value seen for a *p-*type only generator. Future challenges include improving the thermoelectric performance of the *n-*type legs and optimizing the electrical contacts, leg dimensions, and heat sinks.

## Data Availability

All data created during this research are openly available from the Swansea University data archive at 10.5281/zenodo.6447783.

## Supporting Information Available

XRD pattern for MA Sn + Se compared to a commercially sourced SnSe powder, SEM images of all samples, thermoelectric characterization data for three thermal cycles, diffusivity data and density values, and raw data from thermoelectric generator testing (PDF)
