# Supplementary material for: Fully 3D Printed Tin Selenide (SnSe) Thermoelectric Generators with Alternating n-Type and p-Type Legs
Source: ACS Appl Energy Mater. 2023 May 3;6(10):5498–507. doi: 10.1021/acsaem.3c00576 (PMC10206617; doi:10.1021/acsaem.3c00576)
Supplement: Supplementary file 1 — ae3c00576_si_001.pdf [file ae3c00576_si_001.pdf]

Supporting Information - Fully Printed 3D Tin Selenide (SnSe) Thermoelectric Generators with Alternating n-type and p-type Legs

Matthew Richard Burton[1], Geraint Howells[1], Shahin Mehraban[2], James D. McGettrick[1], Nicholas Lavery[2] and Matthew J. Carnie[1]*

Dr. M. R. Burton, G. Howells, Dr. J. McGettrick, Prof. M. J. Carnie

[1]SPECIFIC, Materials Research Centre, College of Engineering, Swansea University, Bay Campus, Swansea, SA1 8EN, United Kingdom

E-mail: m.r.burton@swansea.ac.uk

Dr. S. Mehraban, Prof. N. P. Lavery

[2]MACH1, Materials Research Centre, College of Engineering, Swansea University, Bay Campus, Swansea, SA1 8EN, United Kingdom.

Keywords: Thermoelectrics, printed, tin selenide, thermoelectric generator, TEG, n-type

X-Ray Diffraction (XRD)

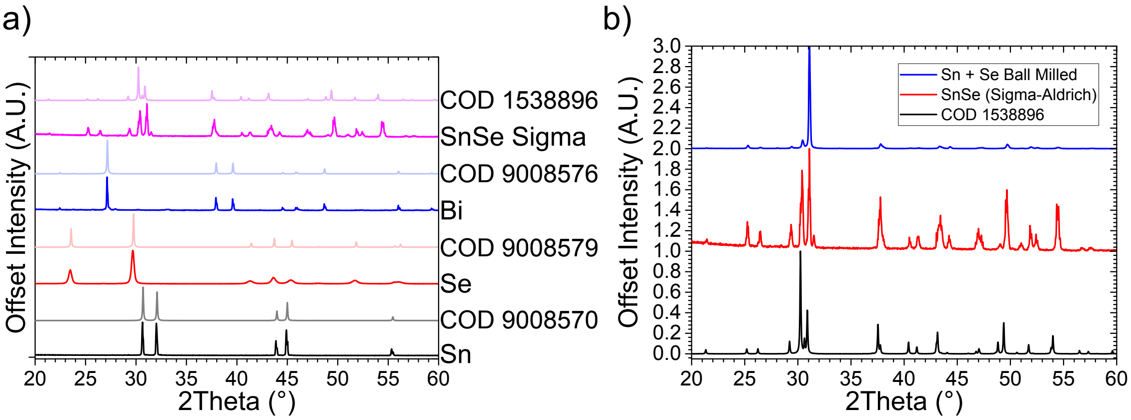

Figure S1. a) XRD patterns of commercial powders used in this work compared with standards from the crystallography open database (COD), b) XRD patterns of the crystallography open database SnSe standard 1538896, commercially sourced SnSe powder and equimolar mechanically alloyed Sn and Se powder.

Table S1. Lattice parameters of all SnSe samples reported in this work and examples from the literature, all samples are assigned to the orthorhombic crystal structure of the Pnma phase.

		Lattice Constant a (Å)	Lattice Constant b (Å)	Lattice Constant c (Å)
Powders	Sn + Se	11.49	4.17	4.45
	Sn + Se + 2% Bi	11.53	4.18	4.45
	Sn + Se + 4% Bi	11.49	4.17	4.44
	Sn + Se + 6% Bi	11.50	4.17	4.44
	Sn + Se + 8% Bi	11.49	4.16	4.44
Printed Samples before cure	Sn + Se	11.53	4.17	4.46
	Sn + Se + 2% Bi	11.51	4.17	4.45
	Sn + Se + 4% Bi	11.51	4.17	4.45
	Sn + Se + 6% Bi	11.51	4.17	4.45
	Sn + Se + 8% Bi	11.53	4.18	4.44
Printed Samples post cure	Sn + Se	11.49	4.17	4.44
	Sn + Se + 2% Bi	11.47	4.15	4.42
	Sn + Se + 4% Bi	11.51	4.17	4.45

	Sn + Se + 6% Bi	11.54	4.17	4.44
	Sn + Se + 8% Bi	11.51	4.18	4.45
	SnSe Sigma	11.51	4.17	4.45
	SnSe COD ID 1538896	11.57	4.19	4.46
	SnSe (Nature, **2014**, 373-377, 508)	11.49	4.14	4.44

Scanning Electron Microscopy (SEM)

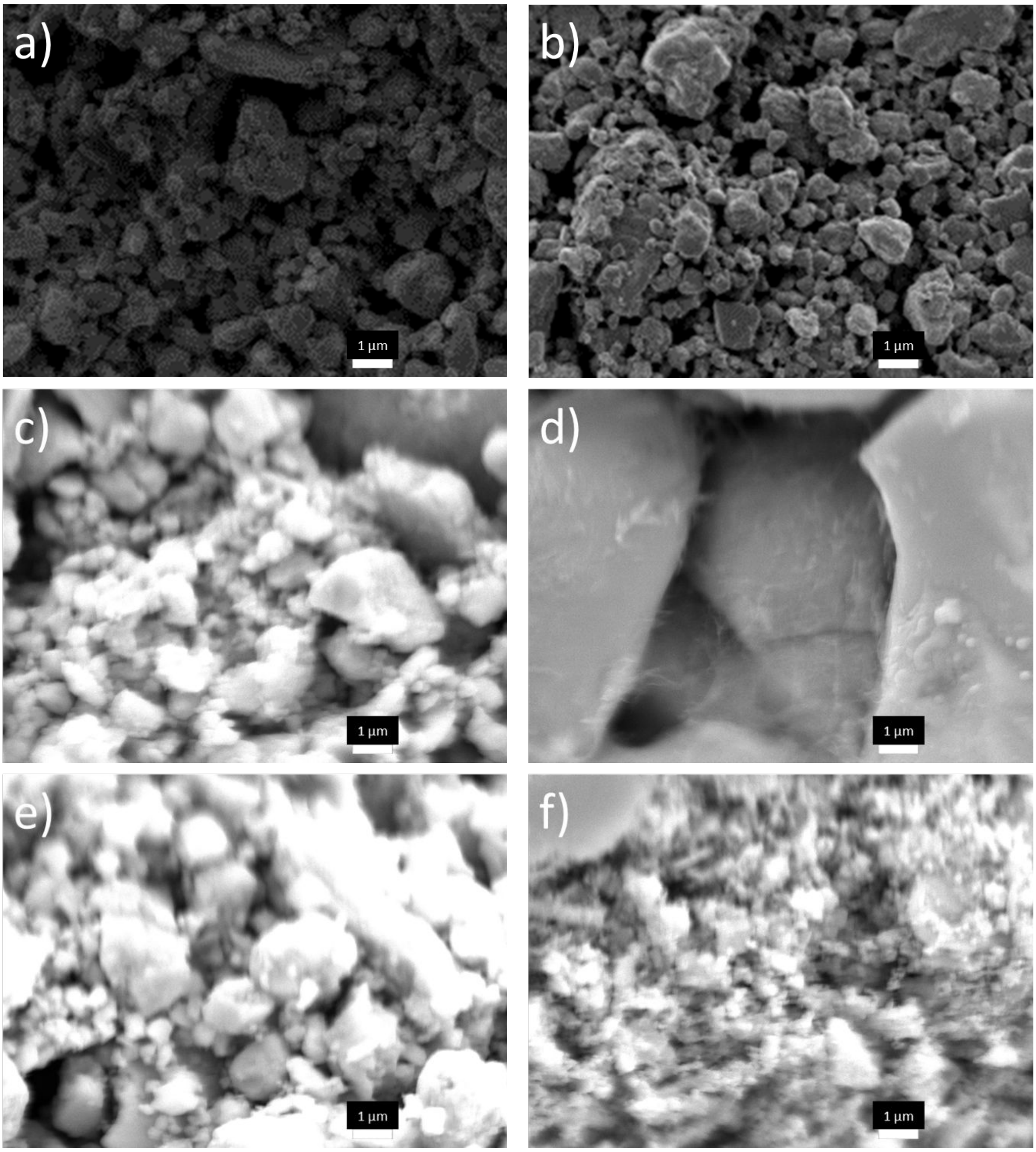
a)
1 µm
b)
1 µm
c)
1 µm
d)
1 µm
e)
1 µm
f)
1 µm

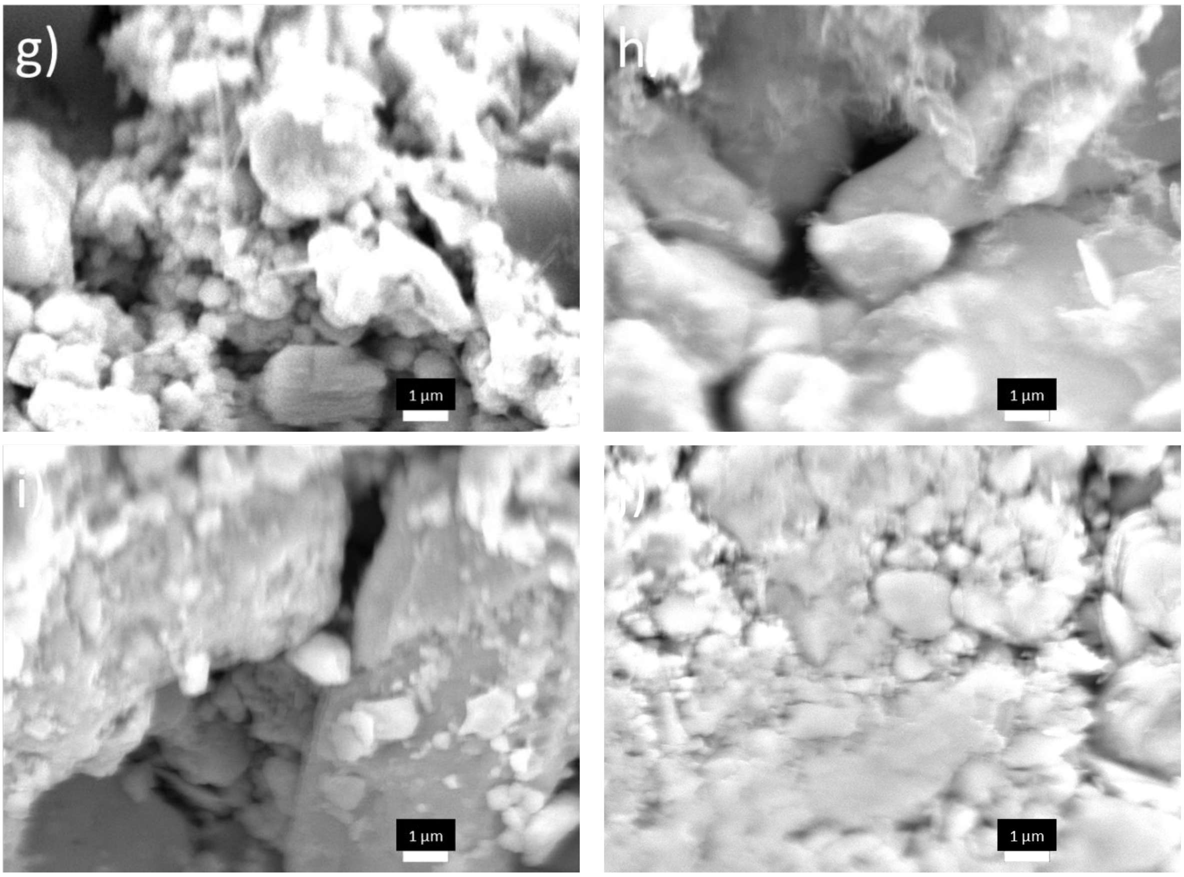

Figure S2. SEM of all printed sample types pre- (a,c,e,g,i) and post- (b,d,f,h,j) cure at 873 K in a He atmosphere: a,b) 0% Bi, c,d) 2% Bi, e,f) 4% Bi, g,h) 6% Bi, i,j) 8% Bi.

<u>Electrical Conductivity (σ)</u>

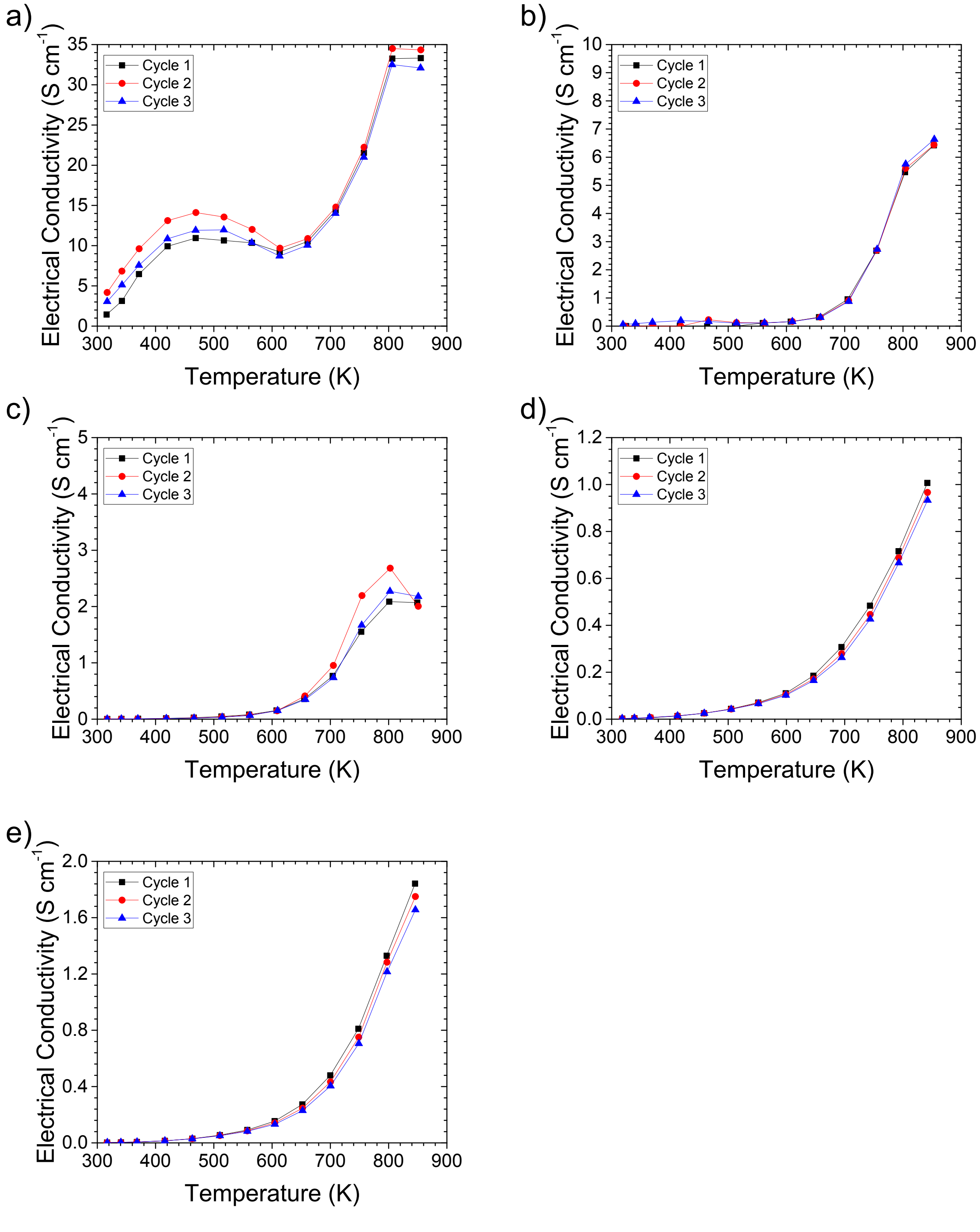

Figure S3. Electrical conductivity through multiple thermal cycles of Bi doped printed SnSe,

a) 0% Bi, b) 2% Bi, c) 4% Bi, d) 6% Bi, e) 8% Bi

Seebeck Coefficient (S)

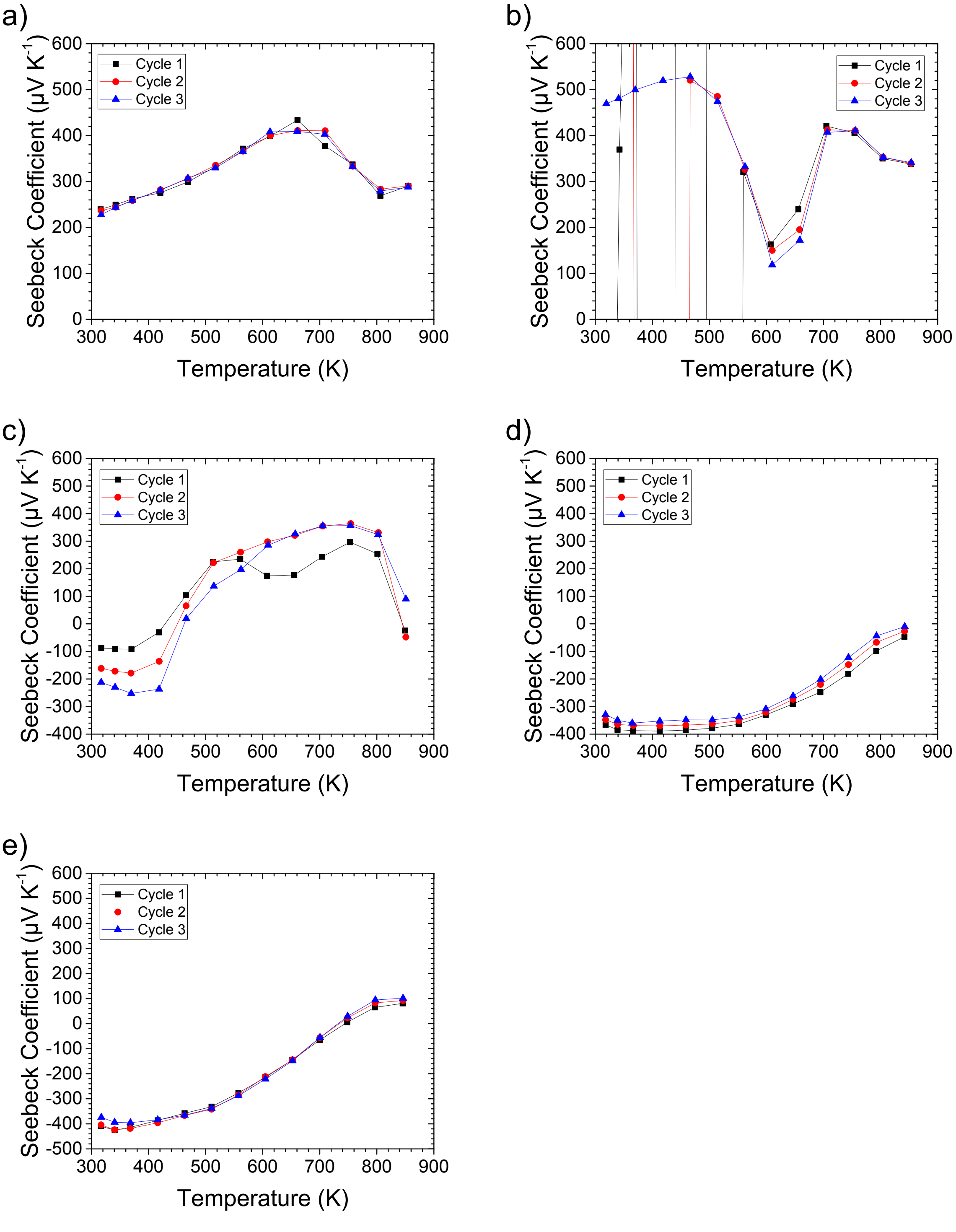

Figure S4. Seebeck coefficient through multiple thermal cycles of Bi doped printed SnSe, a) 0% Bi, b) 2% Bi, c) 4% Bi, d) 6% Bi, e) 8% Bi

Power Factor (PF)

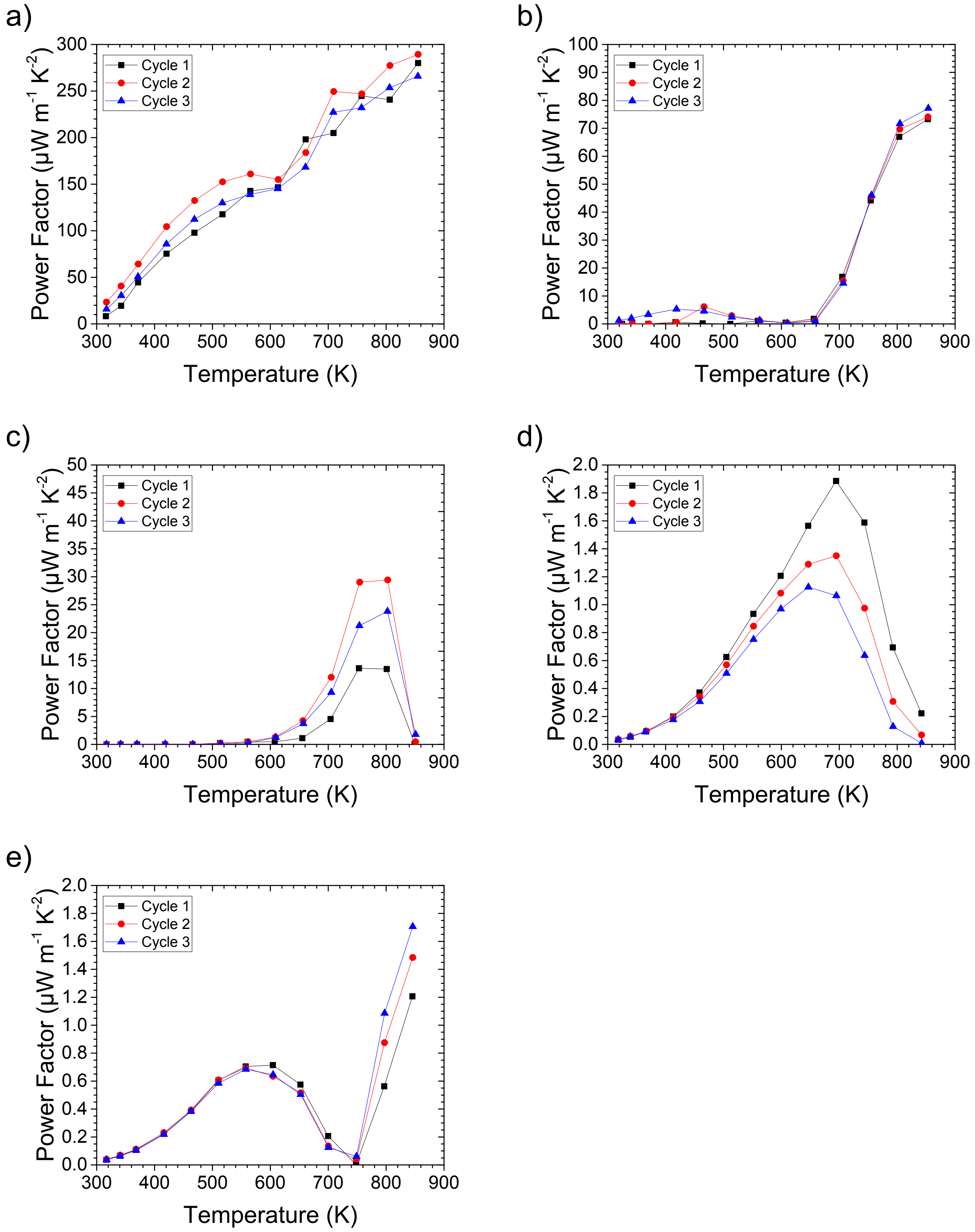

Figure S5. Power factor through multiple thermal cycles of Bi doped printed SnSe, a) 0% Bi, b) 2% Bi, c) 4% Bi, d) 6% Bi, e) 8% Bi

<u>Thermal Conductivity (κ)</u>

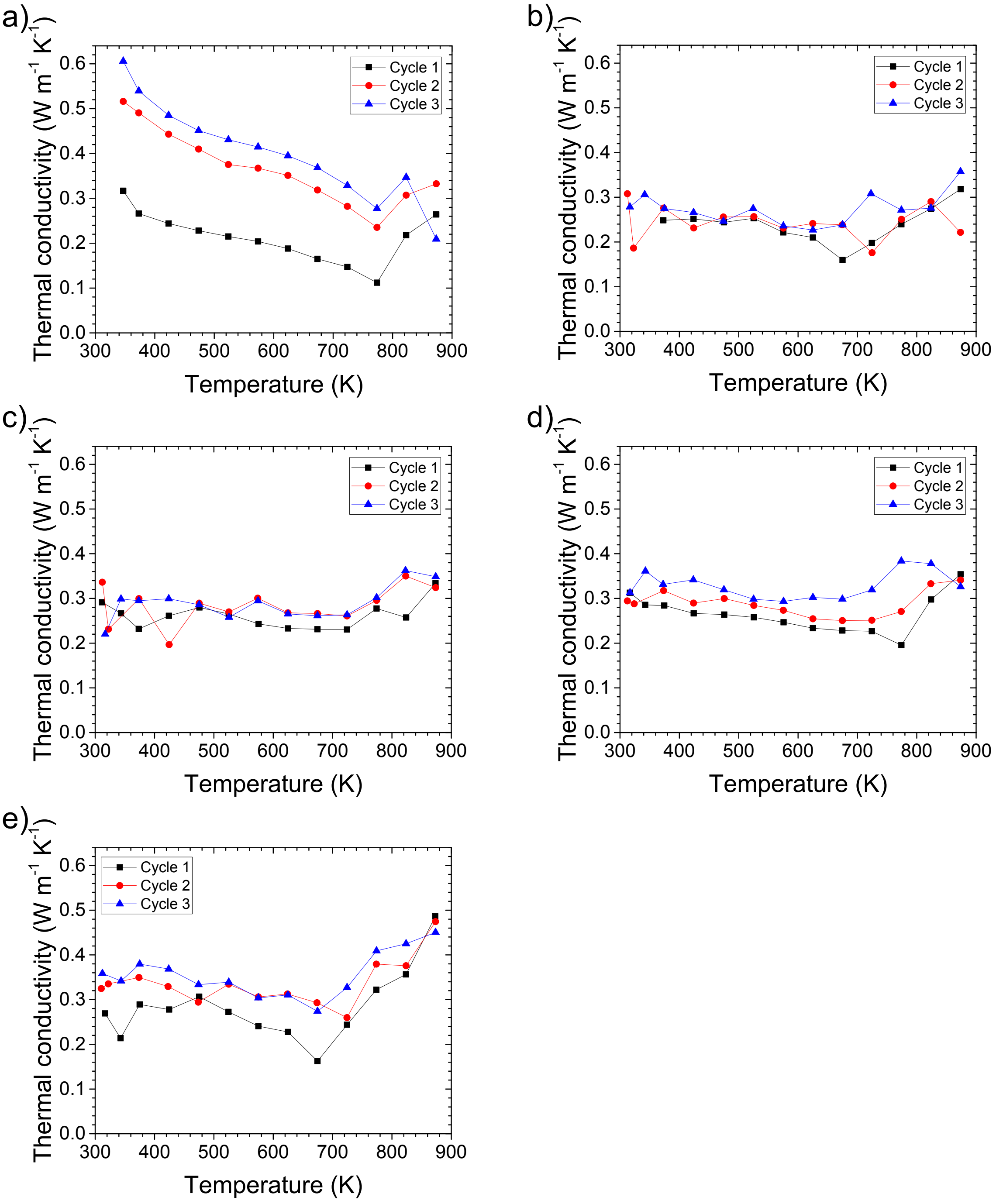

Figure S6. Thermal conductivity through multiple thermal cycles of Bi doped printed SnSe,

a) 0% Bi, b) 2% Bi, c) 4% Bi, d) 6% Bi, e) 8% Bi

zT

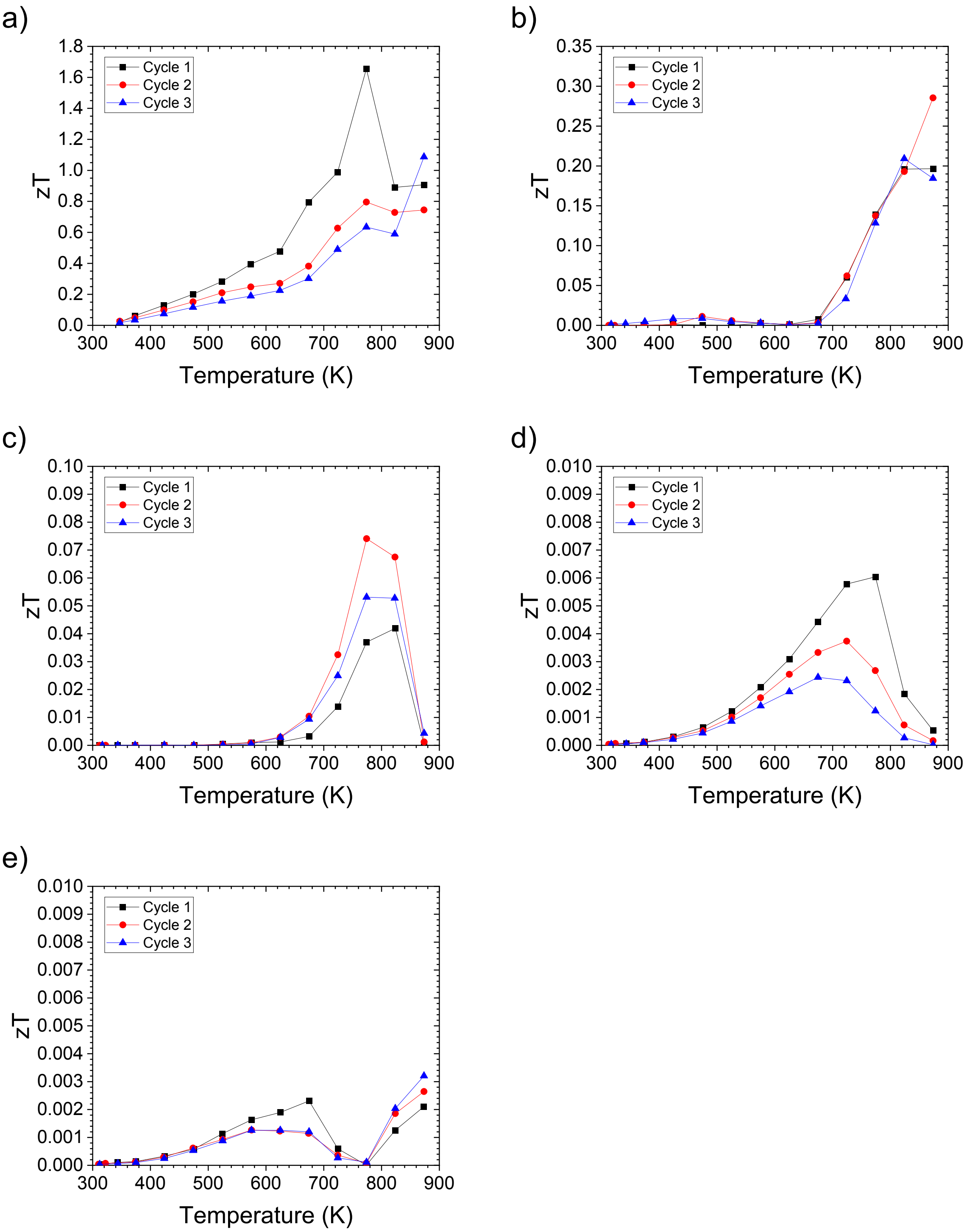

Figure S7. ZT through multiple thermal cycles of Bi doped printed SnSe, a) 0% Bi, b) 2% Bi, c) 4% Bi, d) 6% Bi, e) 8% Bi

Diffusivity

a)
b)
c)
d)
e)

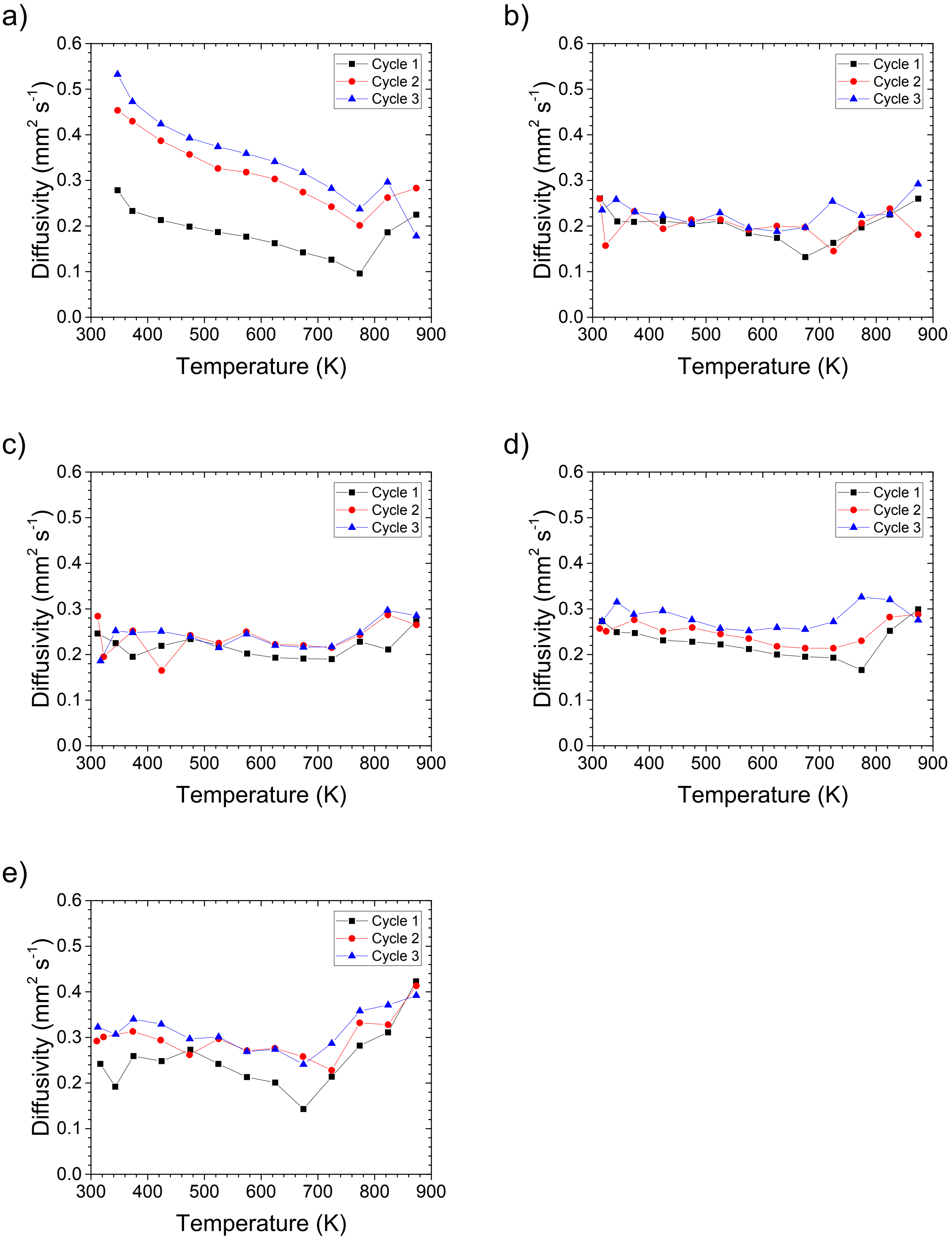

Figure S8. Thermal diffusivity through multiple thermal cycles of Bi doped printed SnSe, a) 0% Bi, b) 2% Bi, c) 4% Bi, d) 6% Bi, e) 8% Bi

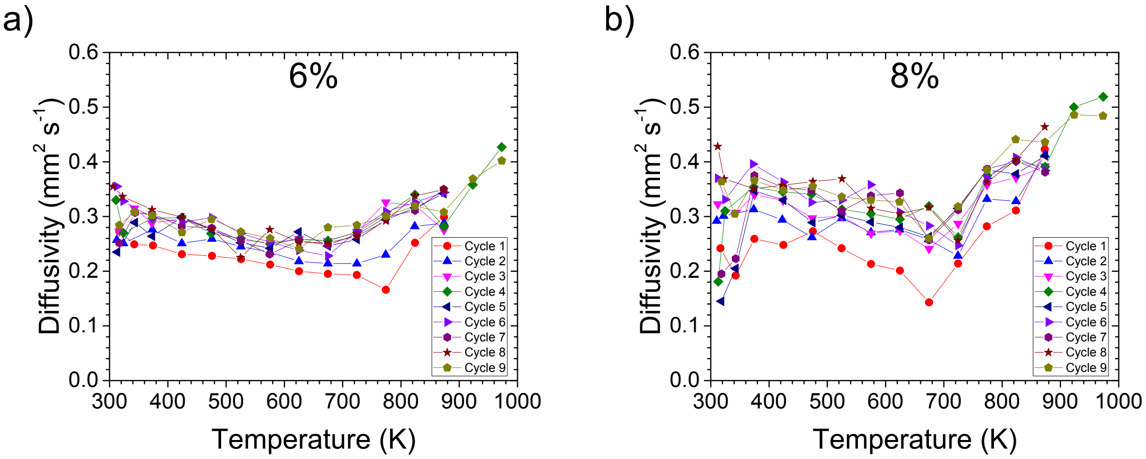

Figure 9. Thermal diffusivity of 6% (a) and 8% (b) Bi doped pseudo-3D printed SnSe after curing at 873 K in He.

<u>Density</u>

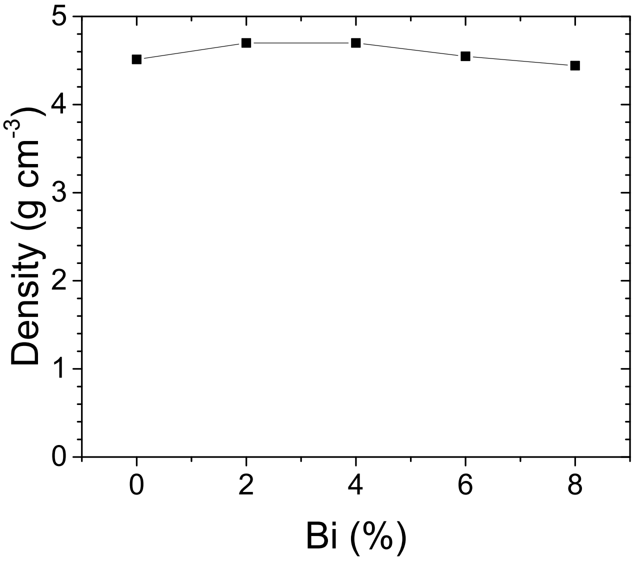

Figure S10. Densities of Bi doped printed SnSe samples determined using the method of hydrostatic weighing.

Altering Binder Concentration

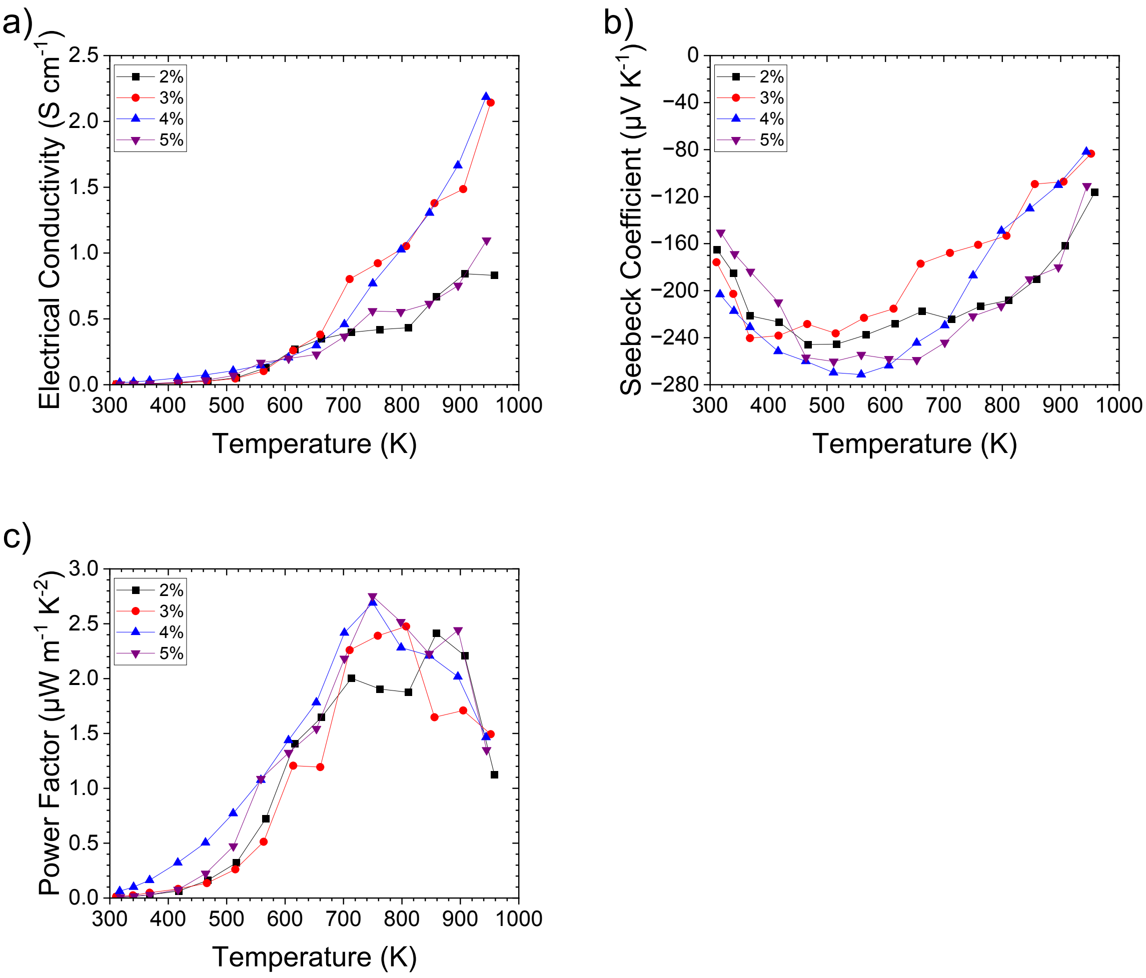

Figure S11. Thermoelectric performance of 2%, 3%, 4% and 5% binder to water ratios of 8% Bi doped pseudo-3D printed SnSe after curing at 873 K in He. The 9th thermal cycle

measurement is shown here for each sample, with the 4% data being the same as seen in Figure 4: a) electrical conductivity, b) Seebeck Coefficient and c) power factor.

Thermoelectric Generator Characterization

Table S1. Performance characterization of all printed 3D thermoelectric generator. Cold side temperature (T_C), hot side temperature (T_H), along with the open-circuit potential (V_0) and short-circuit current (I_0) produced at those temperatures. The output power is calculated assuming maximum power = $V_0 I_0/4$.

T_C (K)	T_H (K)	Potential (mV)	Current (μA)	Power (μW)
296.3	322	0.5	2.00E-04	2.50E-08
304.7	361	33	0.23	0.0019
316	409	53	0.39	0.00517
320.9	455	76	0.61	0.01159
343	499	98	0.9	0.02205
354	538	110	1.6	0.044
372.1	583	112	2.9	0.0812

394.6	608	106	3	0.0795
414	630	117	4.3	0.12578
443.6	645	82	5.2	0.1066
485	707	120	66	1.98
535	744	381	316	30.099
523	774	373	1550	144.5375
453	727	294	812	59.682
420	684	276	279.3	19.2717
384	627	363	68	6.171
356	585	255	75	4.78125
355	545	218	79	4.3055
344	502	162.6	61.3	2.49184
330.9	449	135.7	33.1	1.12292
318	402	101.2	17.8	0.45034
308.3	371	65.4	9.8	0.16023
